# Supplementary material for: Episodic transport of discrete magma batches beneath Aso volcano
Source: Nat Commun. 2021 Sep 21;12:5555. doi: 10.1038/s41467-021-25883-y (PMC8455576; doi:10.1038/s41467-021-25883-y)
Supplement: Supplementary file 1 — Supplementary Information [file 41467_2021_25883_MOESM1_ESM.pdf]

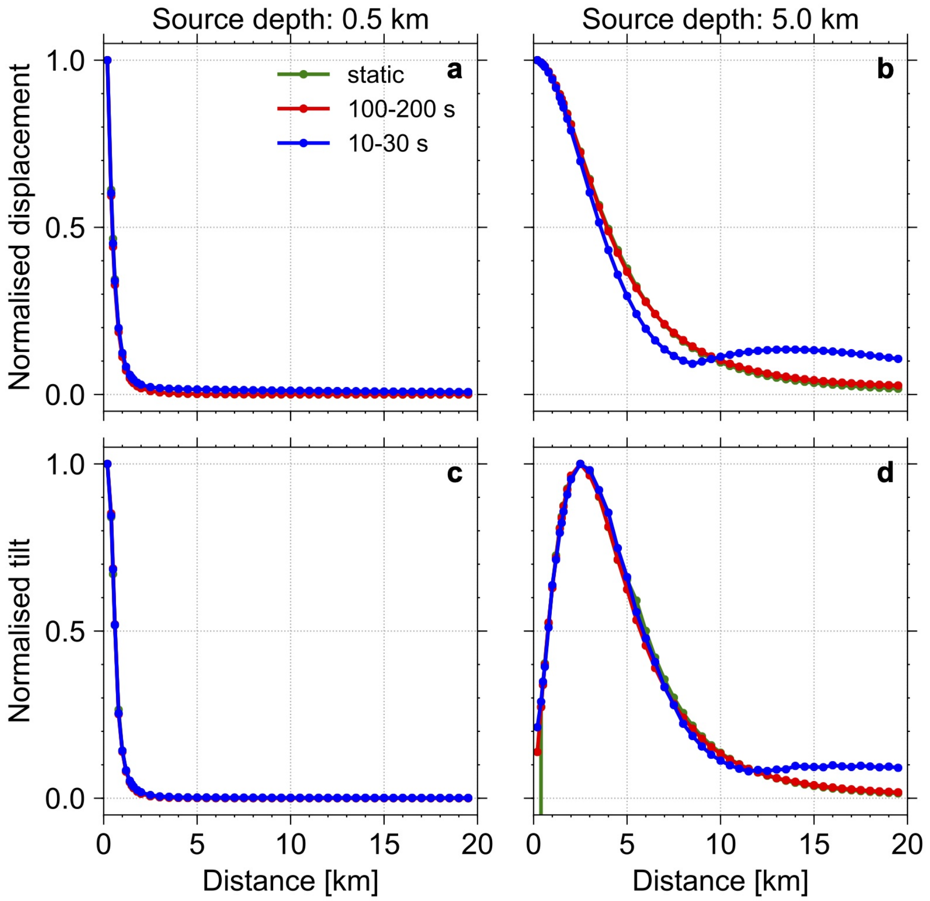

Supplementary Figure 1: **Synthetic amplitude-distance decay against static and filtered waveforms. a-b** The normalized displacement amplitude-distance decay trends with the source depths of 0.5 km and 5.0 km, respectively. **c-d** The normalized tilt amplitude-distance decay trends with the source depths of 0.5 km and 5.0 km, respectively. Static, long-period (10-30 s), and ultra-long-period (100-200 s) amplitude measurements are shown in green, blue and red, respectively.

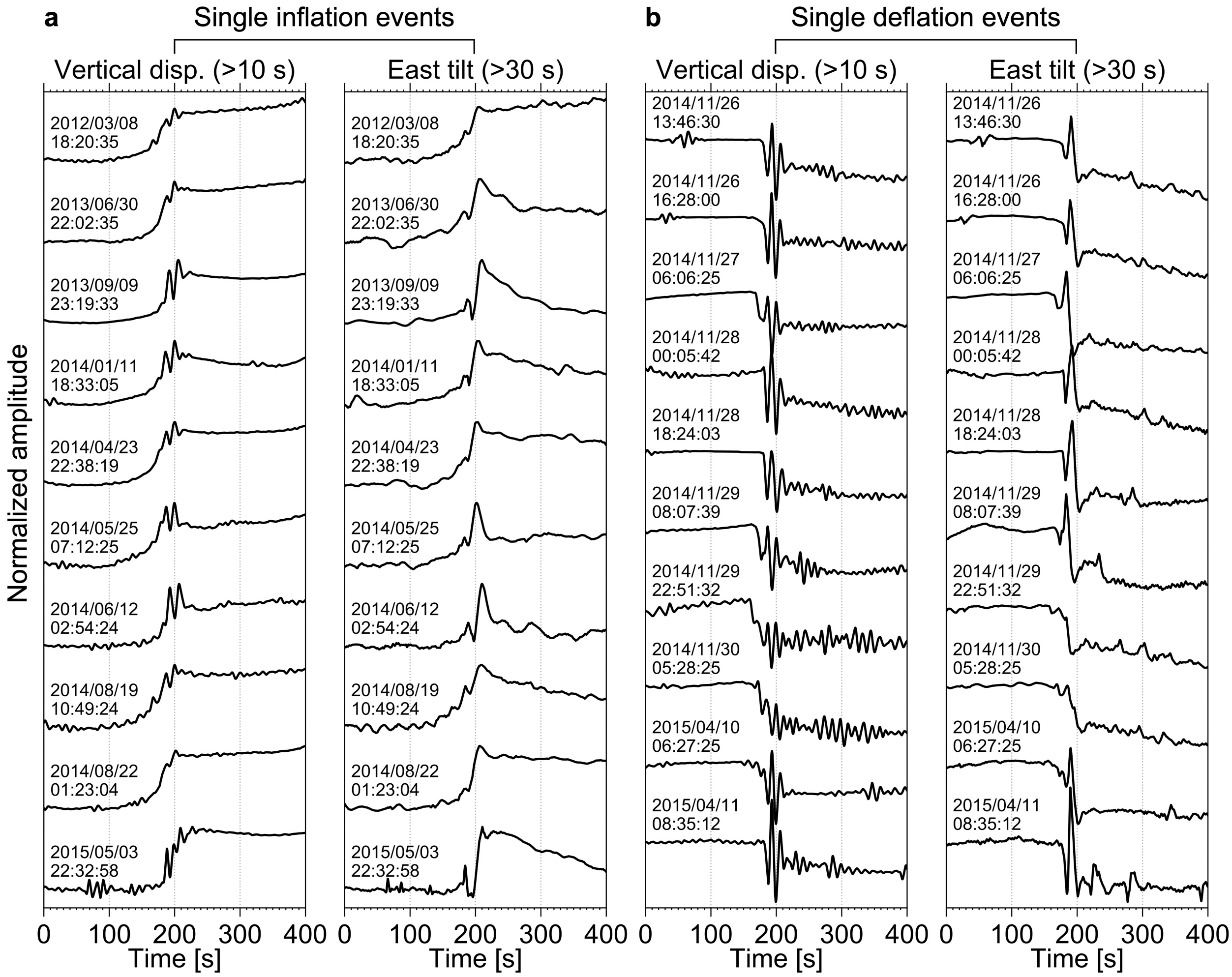

Supplementary Figure 2: **Examples of single-event tilt and displacement waveforms at station N.ASHV.** **a** Low-pass filtered waveforms with the upward displacement and east-down tilt offsets, respectively. **b** Low-passed filtered waveforms with the downward displacement and west-down tilt offsets, respectively. The starting time of each waveform is indicated above each trace.

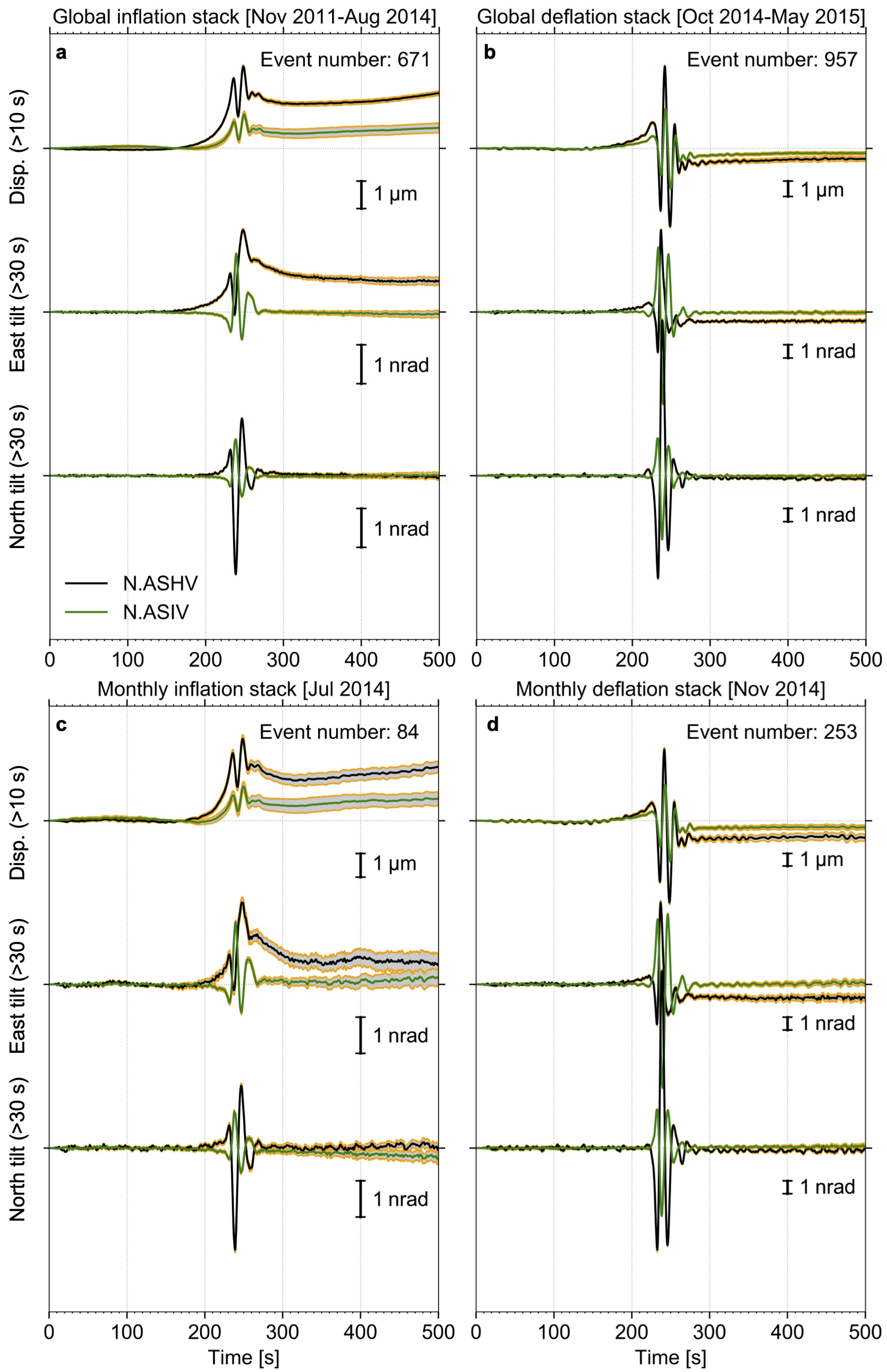

Supplementary Figure 3: **Global and monthly waveform stacks against the highest quality dataset (subset I). a** The global waveform stacks of inflation events during the unrest (November 2011 to August 2014). **b** The global waveform stacks of deflation events (October 2014 to April 2015). **c** The monthly waveform stack of inflation events in July 2014. **d** The monthly waveform stack of deflation events in November 2014. VLP signal is presented in the middle of the time window at 220-270 s. Waveform stacks from stations N.ASHV and N.ASIV are shown in black and green, respectively. The orange shaded strips mark the 99.7% confidence interval estimated by the bootstrapped resampling (ref.36). Displacement and tilt waveform stacks are low-pass filtered at 10 s and 30 s with a 4th-order casual Butterworth filter, respectively. The number of events used in each waveform stack is shown in the upper right.

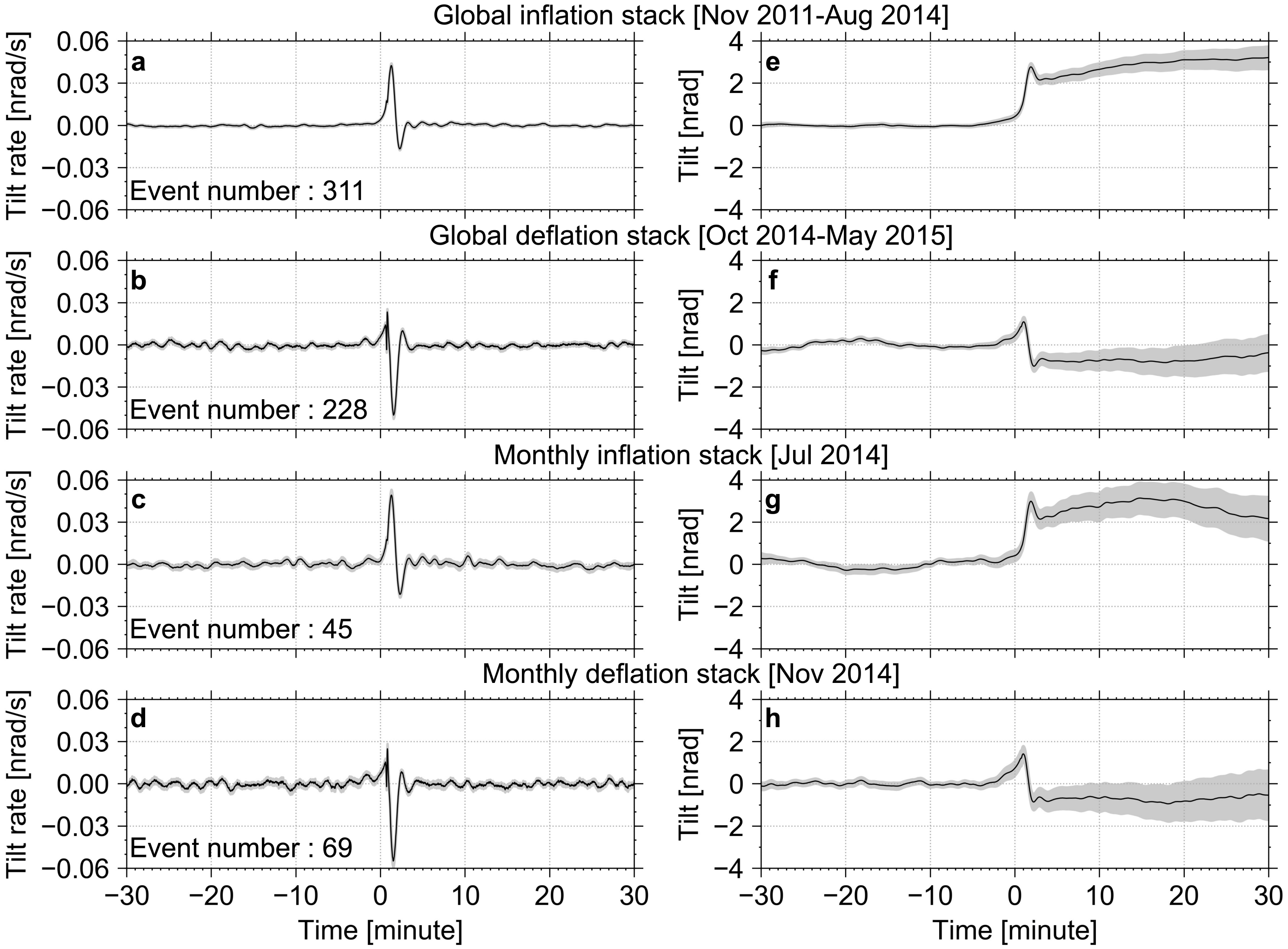

Supplementary Figure 4: **One-hour long global and monthly waveform stacks against the highest quality dataset (subset I).** Same as Supplementary Fig. 2, expect only waveform data associated with isolated VLPs are included in the stack. The number in the left panel indicates the number of the well-isolated VLPs used in the stacks. **a-d** The global and monthly tilt-rate waveform stacks. **e-h** The global and monthly tilt stacks integrated from (a-d), respectively. The trend defined in [-10,-2] minutes of the tilt waveform stacks is used to remove the background trend. The gray shaded region displays the uncertainty at the 99.7% confidence level estimated from the bootstrap resampling (ref.36).

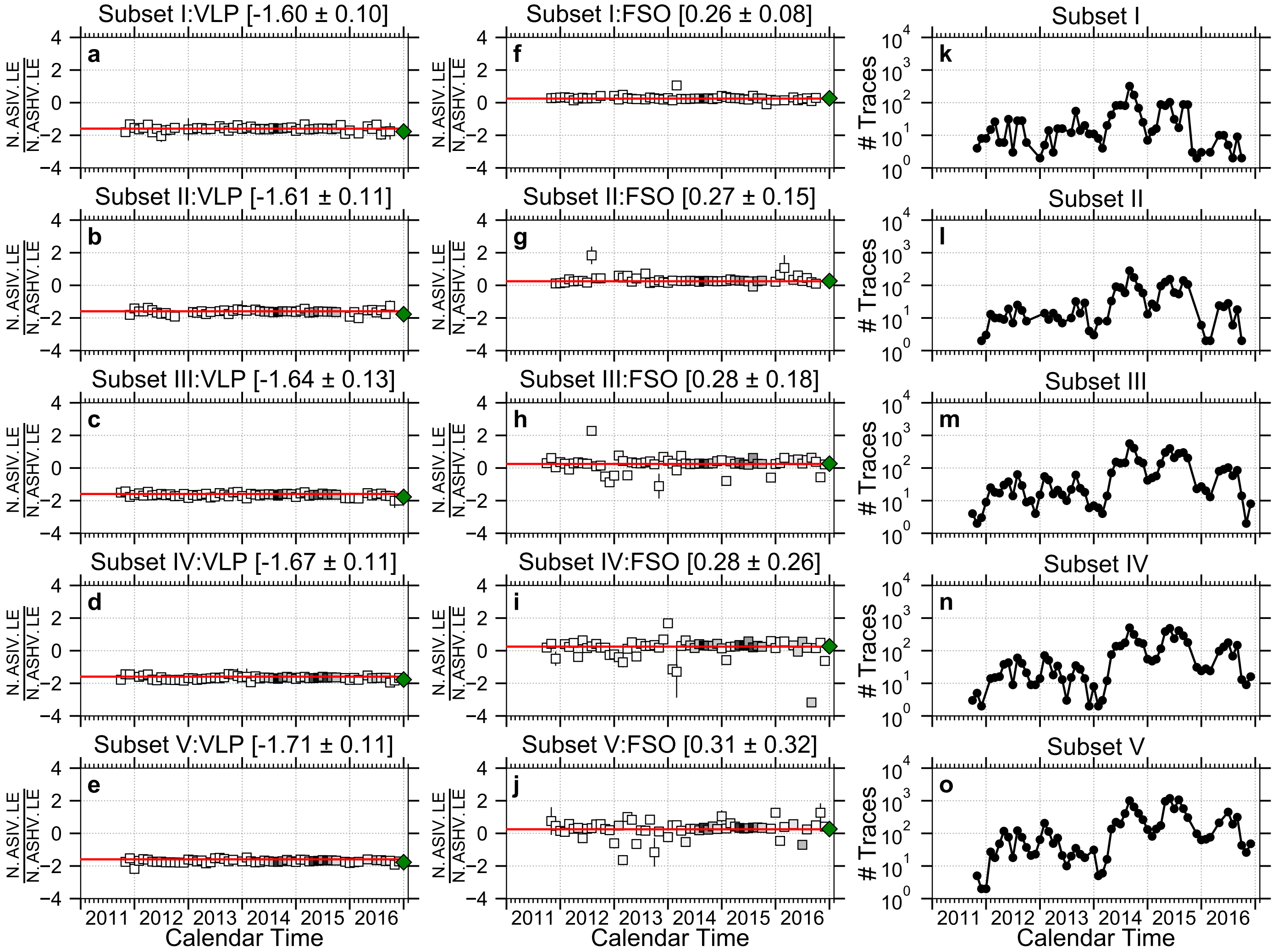

Supplementary Figure 5: **Amplitude ratios of east-west tilts between stations N.ASIV and N.ASHV in the VLP and FSO frequency bands from monthly waveform stacks of inflation events in 2011-2016. a-e** The amplitude ratio in the VLP frequency band (10-30 s) against the five subsets, respectively (the subset I:the highest quality; the subset V: the lowest quality). Square shows the amplitude ratio measured in each monthly stack. The lightness of the square denotes the number of stacked inflation events in a linear scale (dark: higher number; light: lower number). Error bar marks the uncertainty with a 99.7% confidence level estimated from the bootstrap resampling (ref.36). Red line marks the amplitude ratio measured from the global waveform stack of inflation events (Supplementary Fig. 2a). Green diamond marks the amplitude ratio from Event 1 (Fig.1). **f-j** Same as (a-e), except for the measurements in the filtered-static-offset (FSO) frequency band (100-200 s). **k-o** The number of events used in each monthly stack against the five subsets, respectively. The amplitude ratio in the FSO frequency band becomes more variable when the data quality is low or/and the number of events in the monthly stack is low.

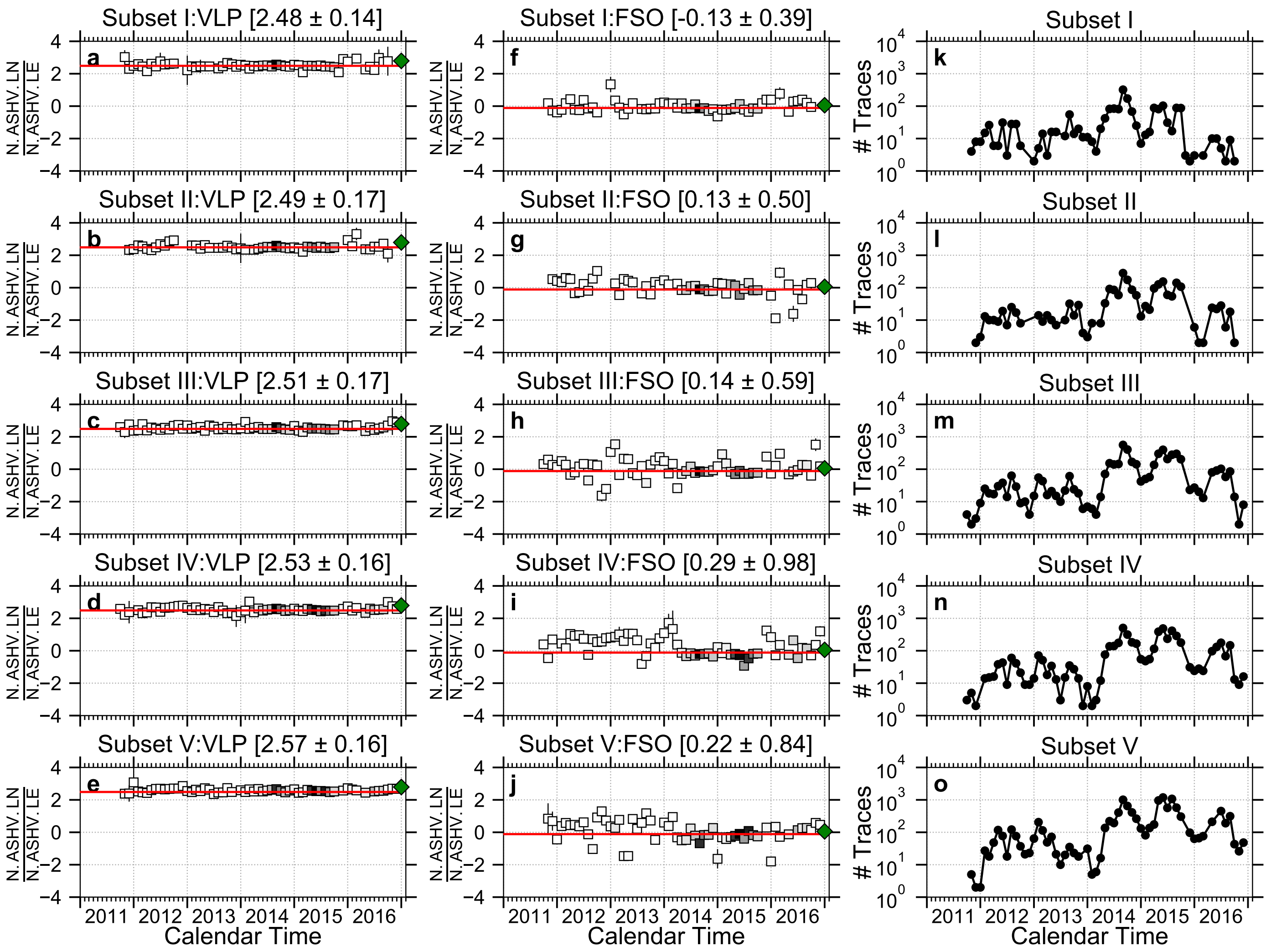

Supplementary Figure 6: **Amplitude ratios between north-south and east-west tilts at station N.ASHV in the VLP and FSO frequency bands from monthly waveform stacks of inflation events in 2011-2016.** Same as Supplementary Fig. 5, except for the amplitude ratio between north-south and east-west tilts at station N.ASHV.

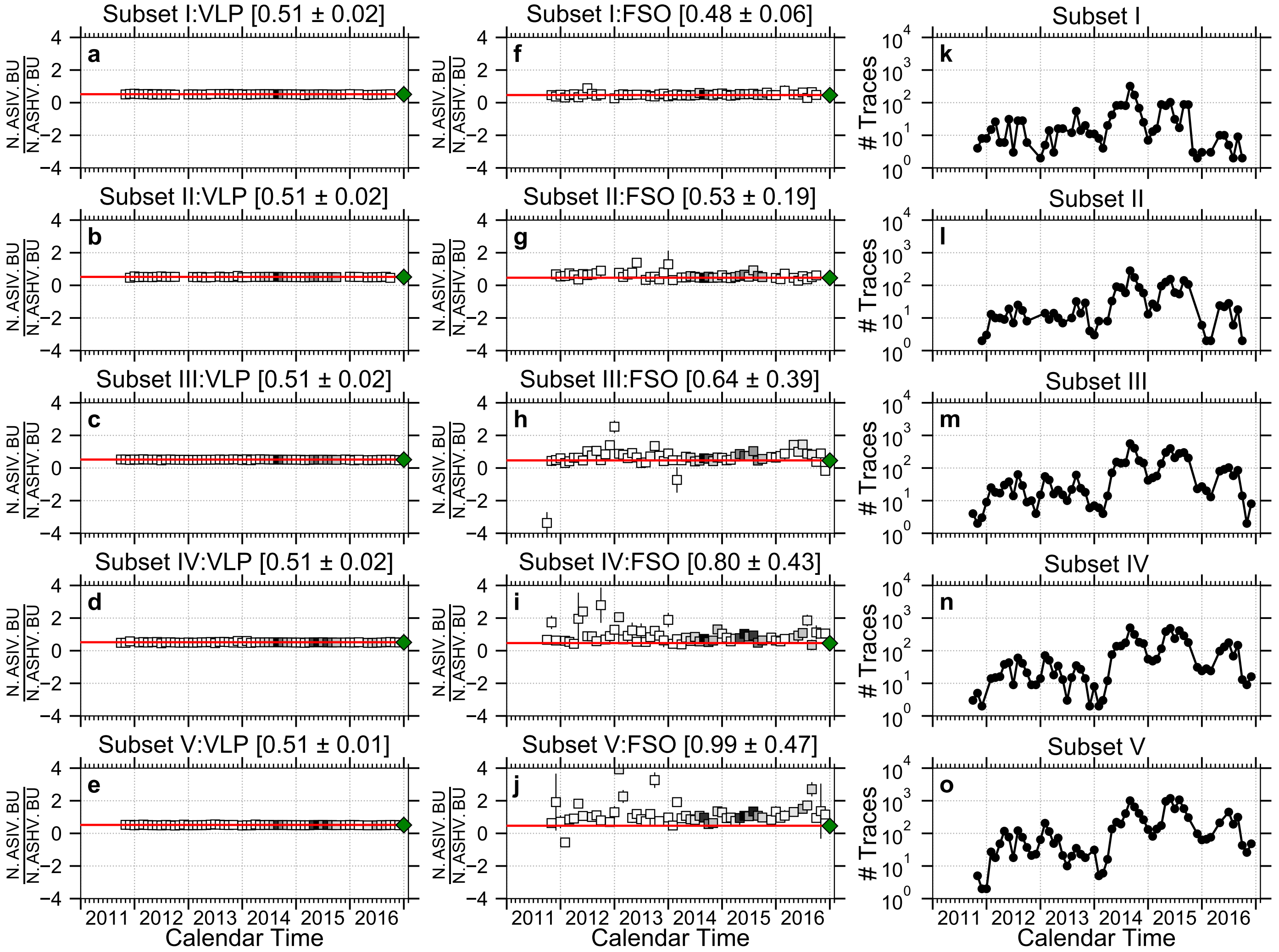

Supplementary Figure 7: **Amplitude ratios of vertical displacements between stations N.ASIV and N.ASHV in the VLP and FSO frequency bands from monthly waveform stacks of inflation events in 2011-2016.** Same as Supplementary Fig. 5, except for the amplitude ratio of vertical displacements between stations N.ASIV and N.ASHV.

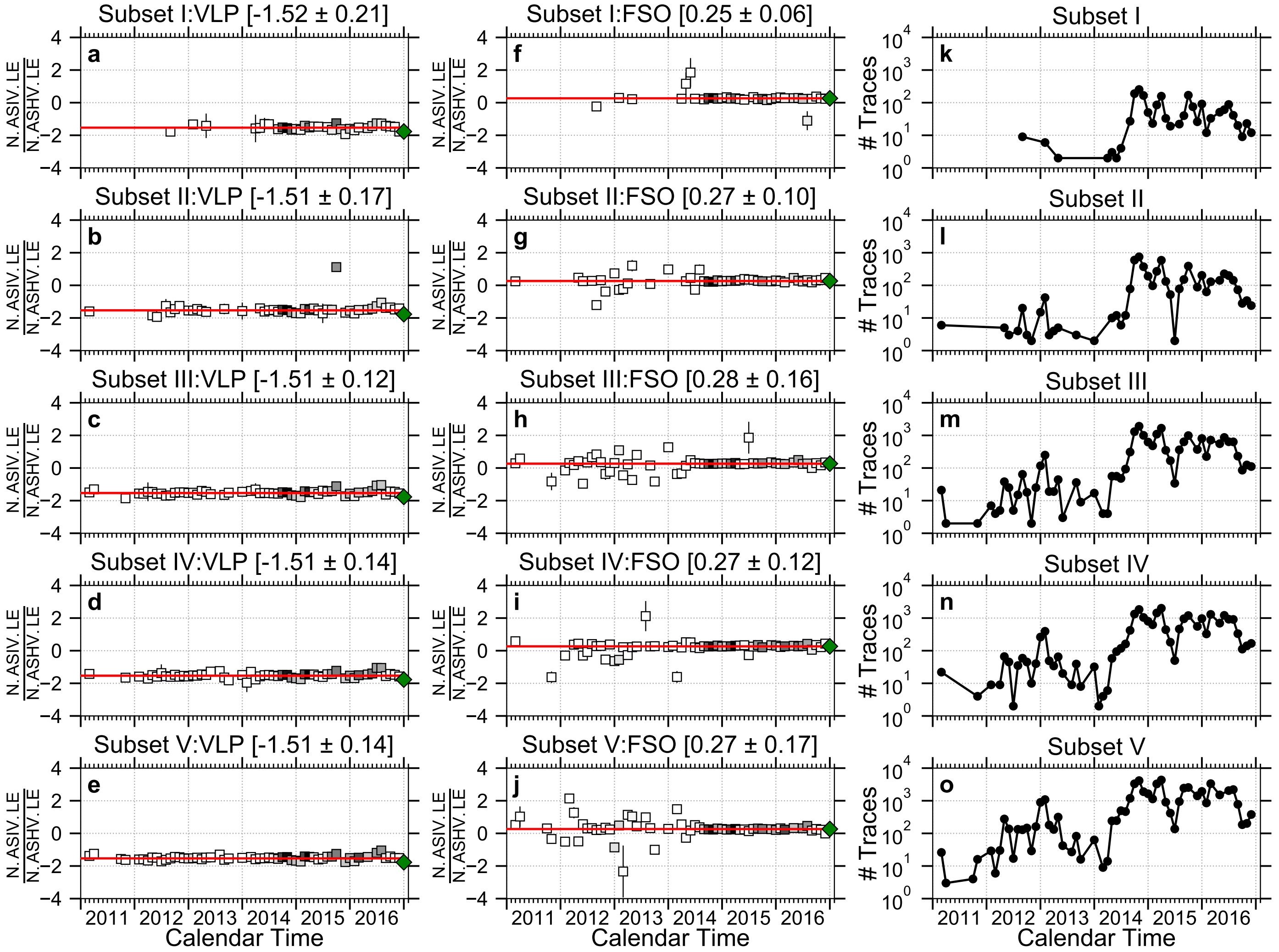

Supplementary Figure 8: **Amplitude ratios of east-west tilts between stations N.ASIV and N.ASHV in the VLP and FSO frequency bands from monthly waveform stacks of deflation events in 2011-2016.** Same as Supplementary Fig. 5, except for the deflation events. Red line marks the amplitude ratio measured from the global waveform stack of deflation events (Supplementary Fig. 2b).

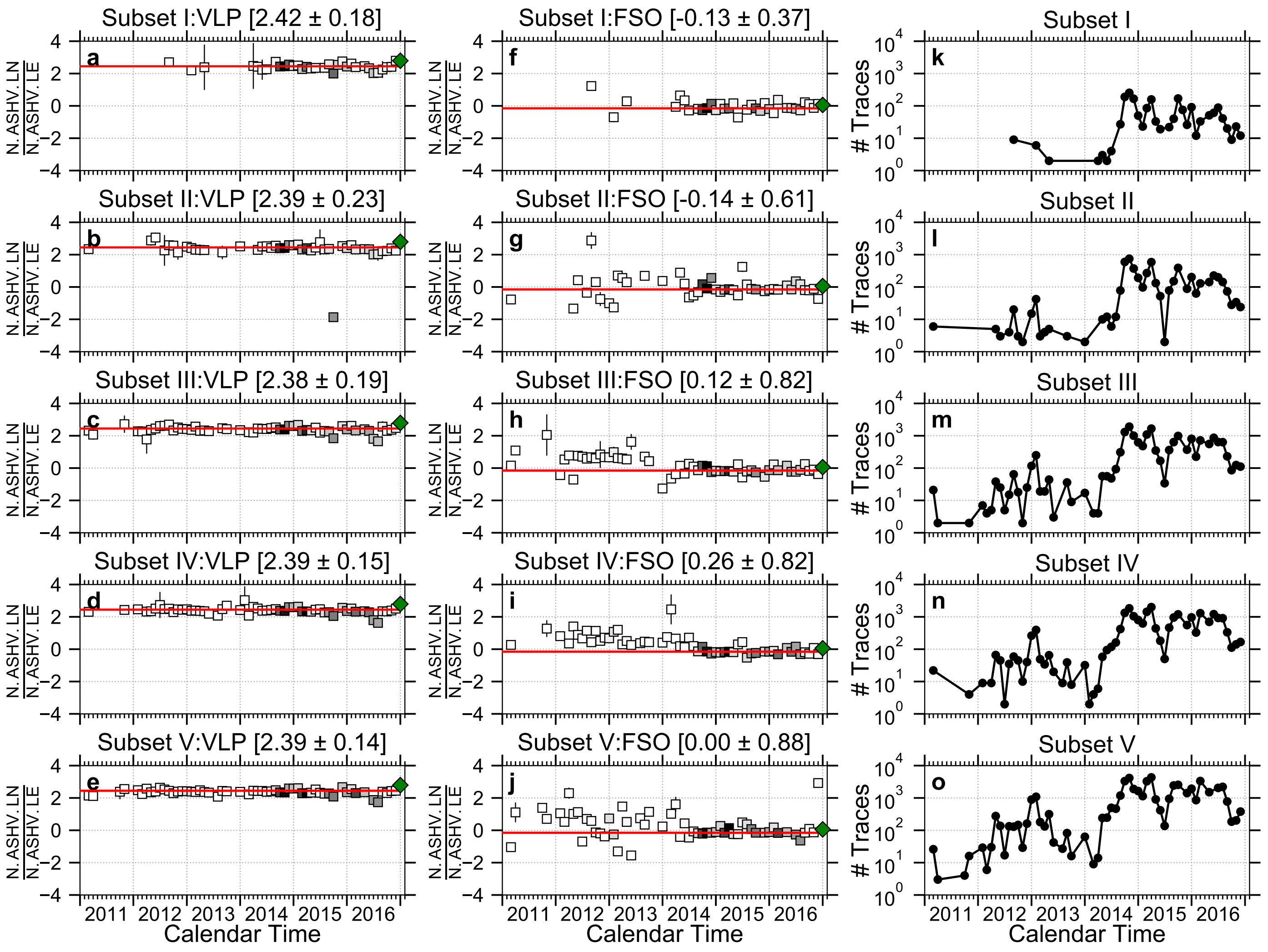

Supplementary Figure 9: **Amplitude ratios between north-south and east-west tilts at station N.ASHV in the VLP and FSO frequency bands from monthly waveform stacks of deflation events in 2011-2016.** Same as Supplementary Fig. 8, except for the amplitude ratio between north-south and east-west tilts at station N.ASHV.

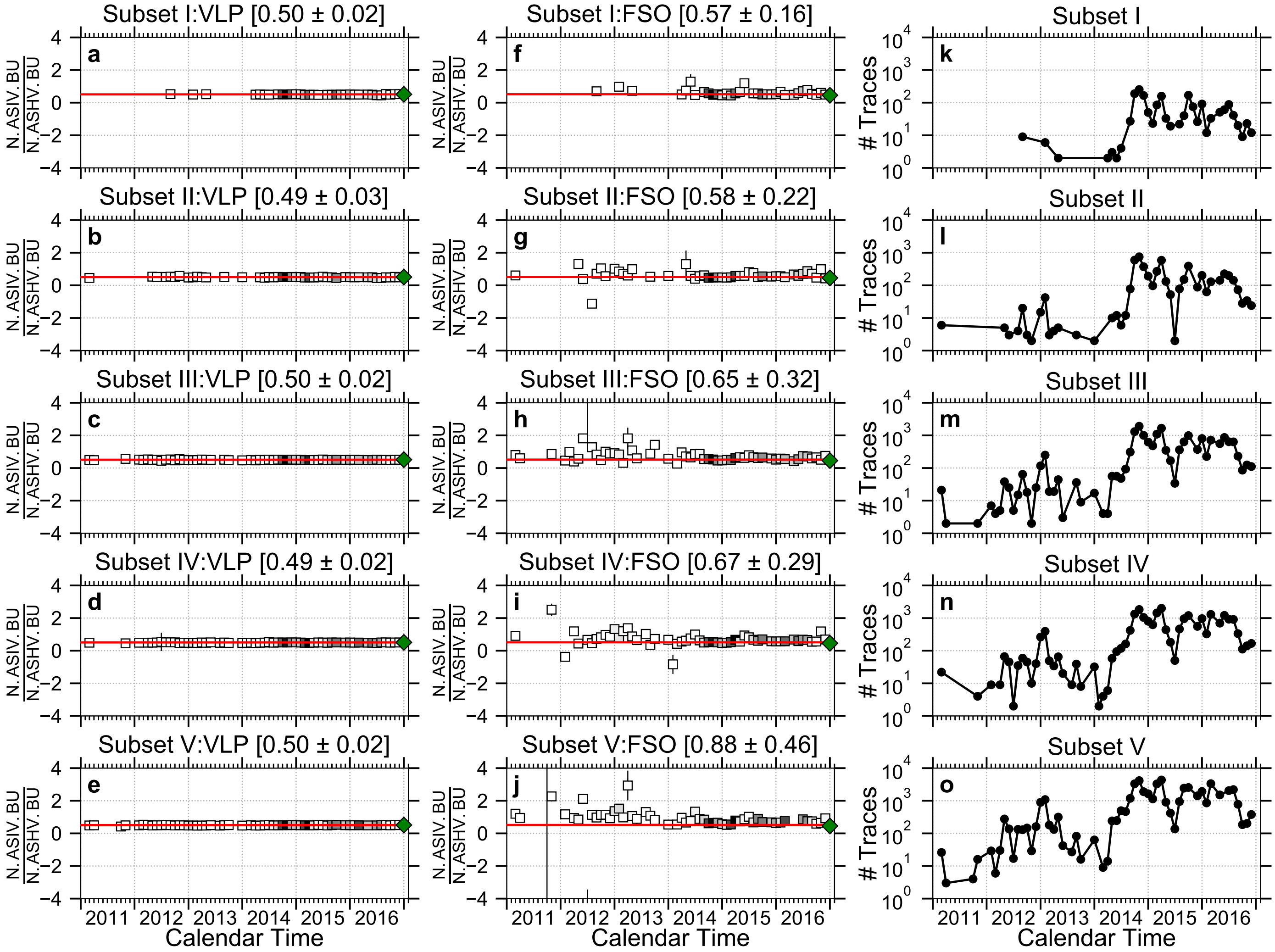

Supplementary Figure 10: **Amplitude ratios of vertical displacements between stations N.ASIV and N.ASHV in the VLP and FSO frequency bands from monthly waveform stacks of deflation events in 2011-2016.** Same as Supplementary Fig. 8, except for the amplitude ratio of vertical displacements between stations N.ASIV and N.ASHV.

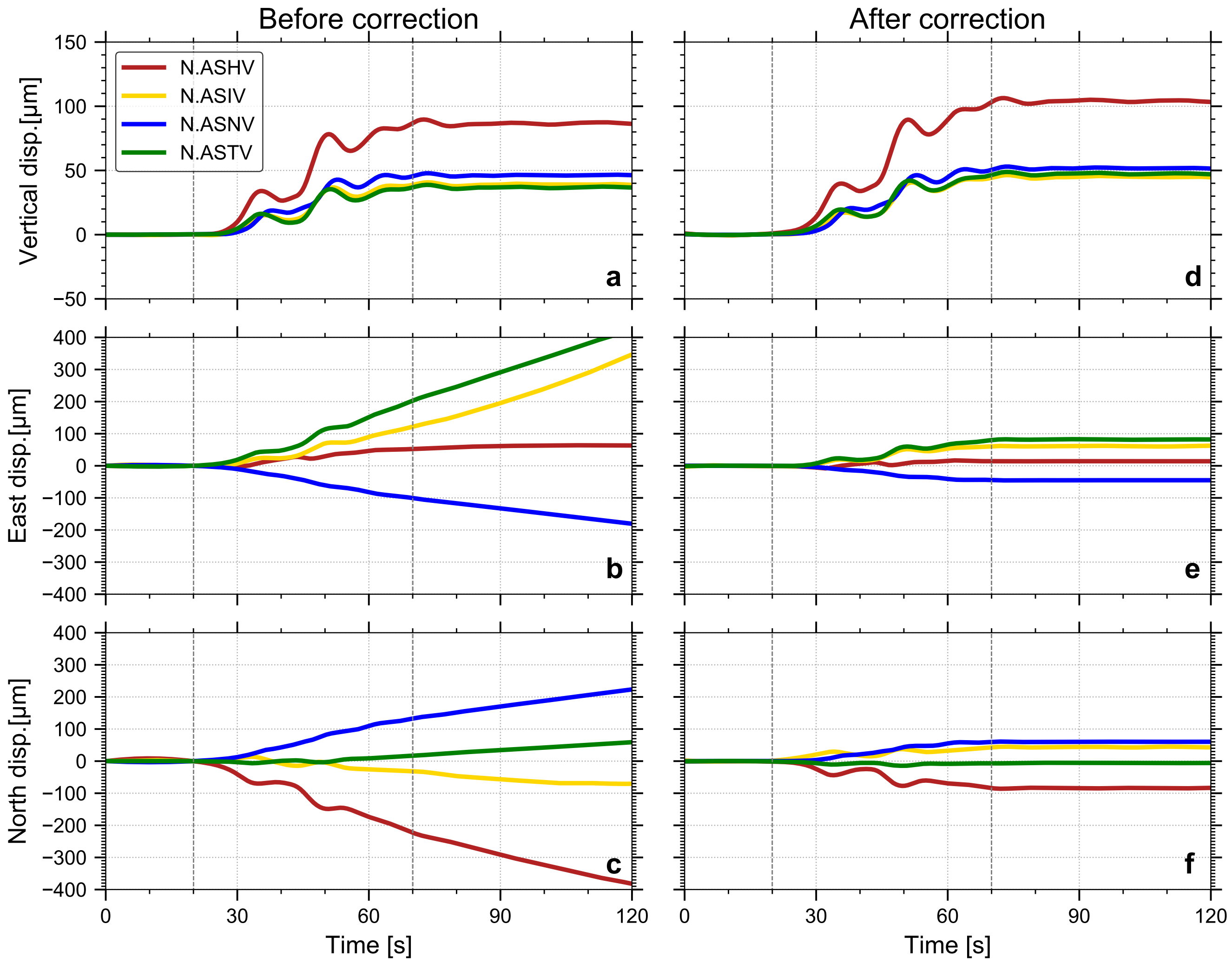

Supplementary Figure 11: **Broadband displacement waveforms before and after the baseline correction.** **a-c** The original broadband displacement waveforms of Event 1 in the vertical, east-west and north-south components at four broadband seismic stations. **d-f** The broadband displacement waveforms after removing the baseline errors. A 4th-order polynomial function is used to estimate the baseline error from the original displacement waveforms in the pre-event and post-event time windows. Vertical dashed lines mark the starting and ending times of the event (see also Method).

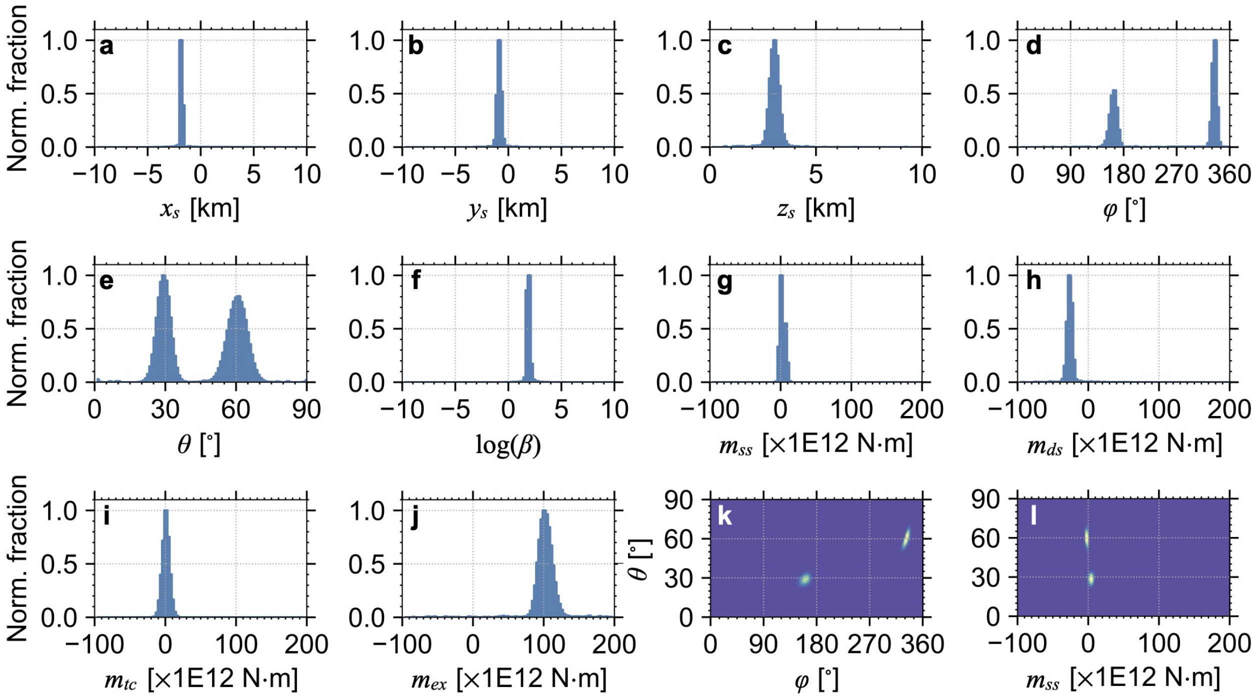

Supplementary Figure 12: **Estimated posterior probability mass functions of the source parameters.** **a-c** The marginal posterior probability mass functions of the 3-D Cartesian location along the eastward (x_s), northward (y_s) and downward (z_s) directions referred to the Naka-dake first crater, respectively. **d-e** The marginal probability mass functions of the strike (ϕ) and dip (θ) angles of the shearing and tensile components. **f** The marginal probability mass function of the logarithm of the regularization parameter. **g-j** The marginal probability mass functions of the moments of the strike-slip (m_{ss}), dip-slip (m_{ds}), tensile-crack (m_{tc}), and isotropic (m_{ex}) components, respectively. **k-l** The joint posterior probability mass functions of the coupled parameters regarding the bimodal functions in (d-e). Two peaks in (d-e) correspond to the orientations of the fault plane and the auxiliary plane.

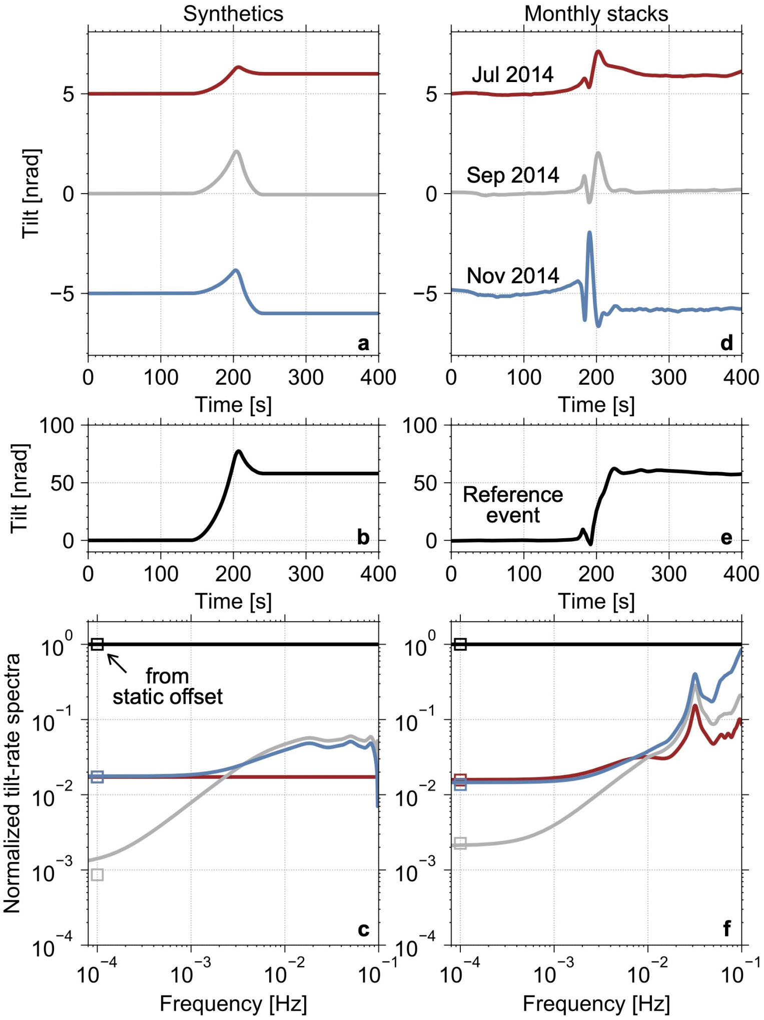

Supplementary Figure 13: **Measurement of tilt offset in the spectra domain.** **a** Synthetic tilt waveforms with the positive (red), null (gray), and negative (blue) offsets of 1, 0 and -1 nrad, respectively. **b** Synthetic tilt waveform with a large offset of 50 nrad. **c** Synthetic tilt-rate amplitude spectra normalized against the amplitude spectrum of (b) at 10,000 s. Squares show the static offsets in (a-b) normalized against the one in (c). **d** Observed monthly tilt waveform stacks with the positive (red), near-null (gray), and negative (blue) offsets, respectively. **e** Observed tilt waveform of the reference event. **f** Observed tilt-rate amplitude spectra normalized against the amplitude spectrum of (e) at 10,000 s. Squares show the static offsets in (d-e) normalized against the one in (e). These examples show that the amplitude plateau of the tilt-rate waveform at 10,000 s provides a reasonable estimate of the static offset.

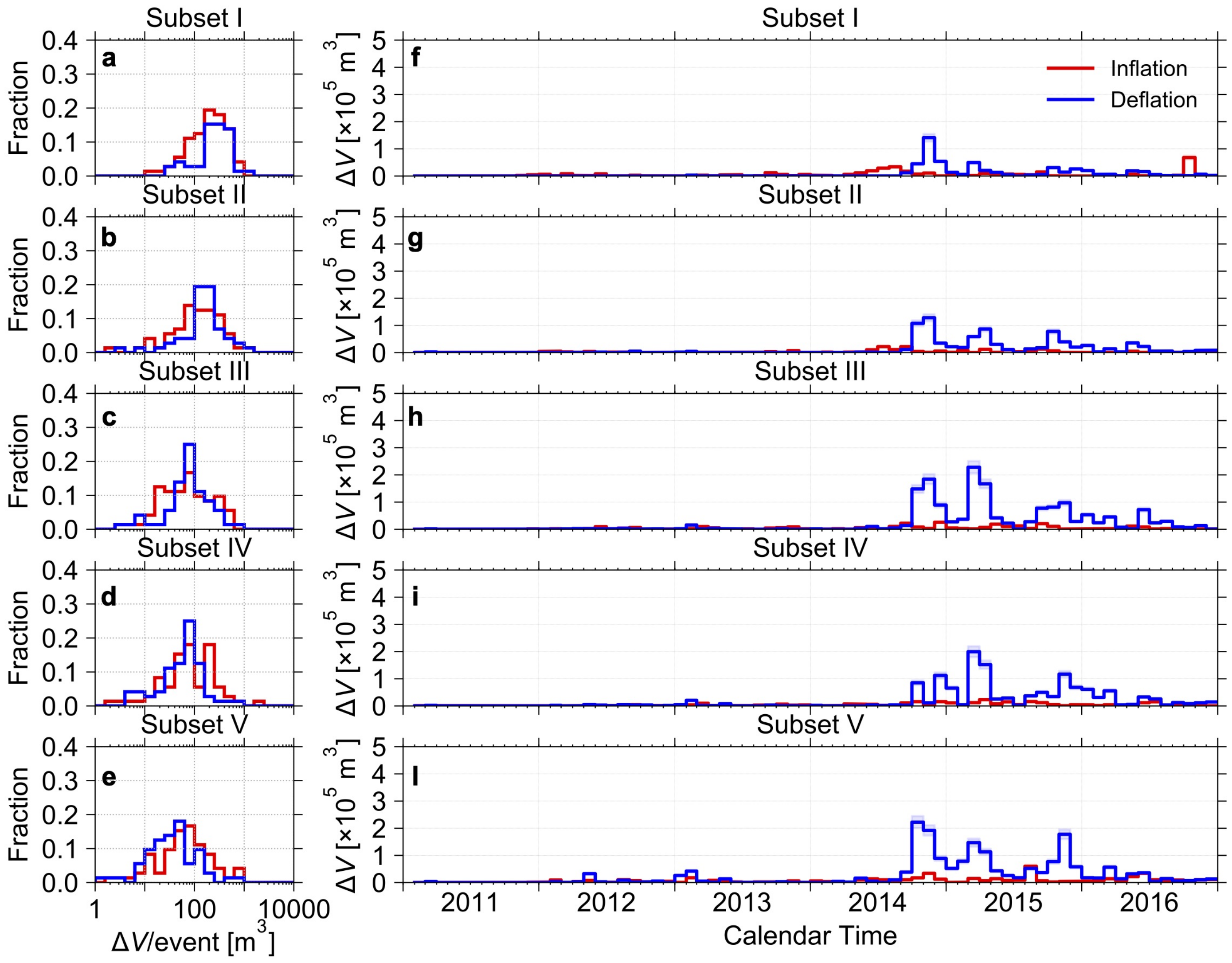

Supplementary Figure 14: **Monthly volume changes in the five subsets.** **a**-**e** Histograms of the volume change per event estimated from the monthly tilt waveform stacks in the five subsets, respectively (see also Methods). Results from the monthly inflation and deflation waveform stacks are shown in red and blue, respectively. **f**-**l** Same as (a-e), except for the monthly volume changes. The shaded region displays the uncertainty at the 99.7% confidence level estimated from the bootstrap resampling (ref.36).

Supplementary Table 1: **Classifications of the ultra-long-period detections against the VLP catalog.**

Subset	Definition	Number	Percentage
I	CC$\geq$0.68 and SNR$\geq$6.9	5562	2.7%
II	CC$\geq$0.58 and SNR$\geq$5.3 (excluding I)	11416	5.5%
III	CC$\geq$0.42 and SNR$\geq$3.7 (excluding I-II)	35766	17.1%
IV	CC$\geq$0.30 and SNR$\geq$2.8 (excluding I-III)	45964	22.0%
V	CC$<$0.30 or SNR$<$2.8	109809	52.7%

Supplementary Table 2: **Summary of estimated source parameters for the reference event.**

Polynomial order	x_s	y_s	z_s	ϕ	θ	m_{ss}	m_{ds}	m_{tc}	m_{ex}	f_E	f_N	f_U	ΔV_m	M_I:M_D	M_w	γ	δ
		$[km]$		[°]			$[\times 10^{14} N \cdot m]$				$[\times 10^{8} N]$		$[m^3]$			[°]	
3	-2.33	-0.64	2.41	324	48	0.007	-0.258	-0.173	1.006				15802	3.2:1	3.3	13.2	72.6
	±0.42	±0.47	±0.23	±37	±37	±0.016	±0.074	±0.062	±0.205				±6524				
4	-1.96	-1.02	2.98	331	60	-0.021	-0.283	-0.002	0.992				25838	4.3:1	3.3	0.1	76.8
	±0.22	±0.27	±0.23	±18	±24	±0.014	±0.080	±0.050	±0.201				±6070				
5	-1.97	-1.08	2.84	334	60	-0.014	-0.286	0.019	0.903				24774	4.0:1	3.3	-1.5	75.9
	±0.68	±0.56	±0.24	±40	±45	±0.016	±0.085	± 0.049	±0.275				±7781				
6	-2.05	-0.35	3.55	313	48	0.003	-0.395	-0.138	1.583				33014	4.1:1	3.4	7.4	76.4
	±0.55	±0.62	±0.27	±44	±43	±0.013	±0.158	±0.068	±0.374				±10619				
4	-1.97	-1.14	3.01	327	58	-0.015	-0.275	-0.011	0.966	-0.005	-0.005	-0.005	24610	4.2:1	3.3	0.9	76.7
	±0.60	±0.74	±0.24	±25	±24	±0.047	±0.199	±0.063	±0.279	±2.381	±1.758	±2.175	±8240				

Note: x_s, y_s and z_s are the 3-D Cartesian location of the preferred source along the eastward, northward and downward directions referred to the Nakadake first crater, respectively; ϕ and θ are the strike and dip angles of the shearing and tensile components, respectively; m_{ss}, m_{ds}, m_{tc} and m_{ex} are the moments of strike-slip, dip-slip, tensile crack and explosion, respectively; f_E, f_N and f_U are the magnitude of singe force along the eastward, northward and upward directions, respectively; ΔV_m is the equivalent Mogi volume change; M_I and M_D are the isotropic and deviatoric scalar seismic moments of the reconstructed moment tensor (ref.31), respectively; M_w is the moment magnitude; γ and δ are the longitude and latitude with pole (1, 1, 1) for the fundamental lune (ref.80), respectively. The uncertainty is defined at a confidence level of 68% ($\pm 1\sigma$).
